# Supplementary material for: An effort-based social feedback paradigm reveals aversion to popularity in socially anxious participants and increased motivation in adolescents
Source: PLoS One. 2021 Apr 27;16(4):e0249326. doi: 10.1371/journal.pone.0249326 (PMC8078767; doi:10.1371/journal.pone.0249326)
Supplement: S1 Fig — Participants chose the hard task less often as the task progressed. (DOCX) [file pone.0249326.s001.docx]

*S1 Fig.* Task fatigue. Participants chose the hard task less often as the task progressed.
