# Supplementary material for: An effort-based social feedback paradigm reveals aversion to popularity in socially anxious participants and increased motivation in adolescents
Source: PLoS One. 2021 Apr 27;16(4):e0249326. doi: 10.1371/journal.pone.0249326 (PMC8078767; doi:10.1371/journal.pone.0249326)
Supplement: S2 Fig — The difference in hard task choices between high and low social status trials by the LSAS total score. Higher difference scores indicate greater preference for high social status trials. Vertical lines indicate cutoff scores of 47 and 60 on the LSAS-SR. (DOCX) [file pone.0249326.s002.docx]

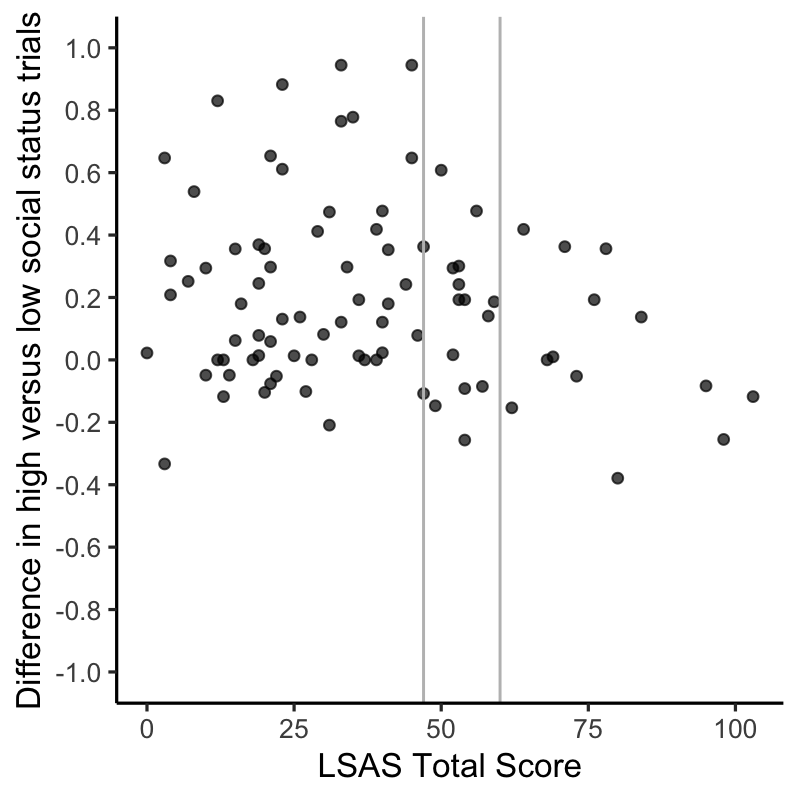


*S2 Fig.* Social anxiety and sensitivity to social status. The difference in hard task choices between high and low social status trials by the LSAS total score. Higher difference scores indicate greater preference for high social status trials. Vertical lines indicate cutoff scores of 47 and 60 on the LSAS-SR.
