# Supplementary material for: An effort-based social feedback paradigm reveals aversion to popularity in socially anxious participants and increased motivation in adolescents
Source: PLoS One. 2021 Apr 27;16(4):e0249326. doi: 10.1371/journal.pone.0249326 (PMC8078767; doi:10.1371/journal.pone.0249326)
Supplement: S1 Table — (DOCX) [file pone.0249326.s003.docx]

**S1 Table.** Social Effort Task Statistics

|  |  | Error df, df | F | p |
| --- | --- | --- | --- | --- |
| **Main Effects** | **Social status** (low/medium/high) | 2, 688 | 48.7 | **< 0.001 ***** |
|  | **Probability** (12%/50%/88%) | 2, 688 | 20.9 | **< 0.001 ***** |
|  | **Age** (adolescents/young adults) | 1, 84 | 4.0 | **0.048 *** |
|  | **Sex** (male/female) | 1, 84 | 10.0 | **0.002 **** |
| **Two-Way Interactions** | **Social status x probability** | 4, 688 | 2.7 | **0.032 *** |
|  | **Social status x age** | 2, 688 | 1.2 | 0.289 |
|  | **Social status x sex** | 2, 688 | 11.4 | **< 0.001 ***** |
|  | **Probability x age** | 2, 688 | 2.5 | 0.084 |
|  | **Probability x sex** | 2, 688 | 2.0 | 0.134 |
|  | **Age x sex** | 1,84 | 0.1 | 0.810 |

Three- and four-way interactions were not significant and dropped from the design.
