# Supplementary material for: An effort-based social feedback paradigm reveals aversion to popularity in socially anxious participants and increased motivation in adolescents
Source: PLoS One. 2021 Apr 27;16(4):e0249326. doi: 10.1371/journal.pone.0249326 (PMC8078767; doi:10.1371/journal.pone.0249326)
Supplement: S2 Table — (DOCX) [file pone.0249326.s004.docx]

**S2 Table.** Social Effort Task Statistics: pairwise comparisons of significant main and interaction effects

|  |  |  | df | *t* | p |
| --- | --- | --- | --- | --- | --- |
| **Social status** |  | Low vs. medium |  | 6.3 | **< 0.001 ***** |
|  |  | Medium vs. high |  | 3.5 | **0.002 **** |
|  |  | Low vs. high |  | 9.7 | **< 0.001 ***** |
| **Probability** |  | 12% vs. 50% |  | 4.3 | **< 0.001 ***** |
|  |  | 50% vs. 88% |  | 2.0 | 0.108 |
|  |  | 12% vs. 88% |  | 6.3 | **< 0.001 ***** |
| **Probability * Social status** | 12% | Low vs. medium |  | 3.5 | **0.001 **** |
|  |  | Med vs. high |  | 1.0 | 0.558 |
|  |  | Low vs. high |  | 4.6 | **< 0.001 ***** |
|  | 50% | Low vs. medium |  | 3.6 | **0.001 **** |
|  |  | Med vs. high |  | 0.8 | 0.709 |
|  |  | Low vs. high |  | 4.4 | **< 0.001 ***** |
|  | 88% | Low vs. medium |  | 3.8 | **< 0.001 ***** |
|  |  | Med vs. high |  | 4.2 | **< 0.001 ***** |
|  |  | Low vs. high |  | 8.0 | **< 0.001 ***** |
|  | Low | 12% vs. 50% |  | 2.5 | **0.030 *** |
|  |  | 50% vs. 88% |  | 0.1 | 0.991 |
|  |  | 12% vs. 88% |  | 2.4 | **0.042 *** |
|  | Med | 12% vs. 50% |  | 2.6 | **0.027 *** |
|  |  | 50% vs. 88% |  | 0.1 | 0.994 |
|  |  | 12% vs. 88% |  | 2.7 | **0.020 *** |
|  | High | 12% vs. 50% |  | 2.4 | **0.049 *** |
|  |  | 50% vs. 88% |  | 3.5 | **0.001 **** |
|  |  | 12% vs. 88% |  | 5.9 | **< 0.001 ***** |
| **Social status x Sex** | Male | Low vs. medium |  | 6.4 | **< 0.001 ***** |
|  |  | Med vs. high |  | 4.2 | **< 0.001 ***** |
|  |  | Low vs. high |  | 10.6 | **< 0.001 ***** |
|  | Female | Low vs. medium |  | 2.6 | **0.027 *** |
|  |  | Medium vs. high |  | 0.8 | 0.679 |
|  |  | Low vs. high |  | 3.4 | **0.002 **** |
|  | Low | Male vs. female |  | 0.2 | 0.857 |
|  | Med | Male vs. female |  | 2.7 | **0.008 **** |
|  | High | Male vs. female |  | 4.9 | **< 0.001 ***** |
