# Supplementary material for: An effort-based social feedback paradigm reveals aversion to popularity in socially anxious participants and increased motivation in adolescents
Source: PLoS One. 2021 Apr 27;16(4):e0249326. doi: 10.1371/journal.pone.0249326 (PMC8078767; doi:10.1371/journal.pone.0249326)
Supplement: S3 Table — (DOCX) [file pone.0249326.s005.docx]

**S3 Table.** Social Effort Task Statistics with continuous age

|  |  | Error df, df | F | p |
| --- | --- | --- | --- | --- |
| **Main Effects** | **Social status** (low/medium/high) | 2, 688 | 4.5 | **0.012 *** |
|  | **Probability** (12%/50%/88%) | 2, 688 | 1.7 | 0.182 |
|  | **Age** | 1, 84 | 4.6 | **0.034 *** |
|  | **Sex** (male/female) | 1, 84 | 0.0 | 0.847 |
| **Two-Way Interactions** | **Social status x probability** | 4, 688 | 2.7 | **0.032 *** |
|  | **Social status x age** | 2, 688 | 0.9 | 0.411 |
|  | **Social status x sex** | 2, 688 | 11.3 | **< 0.001 ***** |
|  | **Probability x age** | 2, 688 | 1.2 | 0.302 |
|  | **Probability x sex** | 2, 688 | 1.9 | 0.146 |
|  | **Age x sex** | 1, 84 | 0.6 | 0.425 |

Three- and four-way interactions were not significant and dropped from the design.
