# Supplementary material for: An effort-based social feedback paradigm reveals aversion to popularity in socially anxious participants and increased motivation in adolescents
Source: PLoS One. 2021 Apr 27;16(4):e0249326. doi: 10.1371/journal.pone.0249326 (PMC8078767; doi:10.1371/journal.pone.0249326)
Supplement: S4 Table — (DOCX) [file pone.0249326.s006.docx]

**S4 Table.** Social Effort Task Statistics with social anxiety

|  |  | Error df, df | F | p |
| --- | --- | --- | --- | --- |
| **Main Effects** | **Social status** (low/medium/high) | 2, 684 | 12.1 | **< 0.001 ***** |
|  | **Probability** (12%/50%/88%) | 2, 684 | 12.5 | **< 0.001 ***** |
|  | **Social anxiety** (non-elevated/elevated) | 1, 684 | 0.1 | 0.821 |
|  | **Age** (adolescents/young adults) | 1, 81 | 2.9 | 0.091 |
|  | **Sex** (male/female) | 1, 81 | 3.6 | 0.062 |
| **Two-Way Interactions** | **Social status x probability** | 4, 684 | 2.7 | **0.030 *** |
|  | **Social status x social anxiety** | 2, 684 | 5.2 | **0.005 **** |
|  | **Social status x age** | 2, 684 | 1.2 | 0.124 |
|  | **Social status x sex** | 2, 684 | 9.4 | **< 0.001 ***** |
|  | **Probability x social anxiety** | 2, 684 | 0.3 | 0.713 |
|  | **Probability x age** | 2, 684 | 2.3 | 0.103 |
|  | **Probability x sex** | 2, 684 | 2.1 | 0.128 |
|  | **Social anxiety x age** | 1, 81 | 0.2 | 0.623 |
|  | **Social anxiety x sex** | 1, 81 | 0.2 | 0.676 |
|  | **Age x sex** | 1, 81 | 0.0 | 0.904 |

Three- and four-way interactions were not significant and dropped from the design.
