# Supplementary material for: An effort-based social feedback paradigm reveals aversion to popularity in socially anxious participants and increased motivation in adolescents
Source: PLoS One. 2021 Apr 27;16(4):e0249326. doi: 10.1371/journal.pone.0249326 (PMC8078767; doi:10.1371/journal.pone.0249326)
Supplement: S5 Table — (DOCX) [file pone.0249326.s007.docx]

**S5 Table.** Social Effort Task Statistics with social anxiety: pairwise comparisons of significant main and interaction effects

|  |  |  | df | *t* | p |
| --- | --- | --- | --- | --- | --- |
| **Social status** |  | Low vs. medium |  | 3.6 | **0.001 **** |
|  |  | Medium vs. high |  | 1.1 | 0.495 |
|  |  | Low vs. high |  | 4.6 | **< 0.001 ***** |
| **Probability** |  | 12% vs. 50% |  | 3.7 | **< 0.001 ***** |
|  |  | 50% vs. 88% |  | 1.1 | 0.506 |
|  |  | 12% vs. 88% |  | 4.8 | **< 0.001 ***** |
| **Probability * Social status** | 12% | Low vs. medium |  | 2.4 | **0.040 *** |
|  |  | Med vs. high |  | 0.1 | 0.998 |
|  |  | Low vs. high |  | 2.4 | **0.047 *** |
|  | 50% | Low vs. medium |  | 2.5 | **0.036 *** |
|  |  | Med vs. high |  | 0.3 | 0.961 |
|  |  | Low vs. high |  | 2.2 | 0.071 |
|  | 88% | Low vs. medium |  | 2.7 | **0.021 *** |
|  |  | Med vs. high |  | 2.7 | **0.018 *** |
|  |  | Low vs. high |  | 5.4 | **< 0.001 ***** |
|  | Low | 12% vs. 50% |  | 2.6 | **0.024 *** |
|  |  | 50% vs. 88% |  | 0.3 | 0.935 |
|  |  | 12% vs. 88% |  | 2.3 | 0.056 |
|  | Med | 12% vs. 50% |  | 2.7 | **0.021 *** |
|  |  | 50% vs. 88% |  | 0.1 | 0.988 |
|  |  | 12% vs. 88% |  | 2.5 | **0.032 *** |
|  | High | 12% vs. 50% |  | 2.6 | **0.038 *** |
|  |  | 50% vs. 88% |  | 2.9 | **0.012 *** |
|  |  | 12% vs. 88% |  | 5.3 | **< 0.001 ***** |
| **Social status x Sex** | Male | Low vs. medium |  | 4.2 | **< 0.001 ***** |
|  |  | Med vs. high |  | 2.0 | 0.104 |
|  |  | Low vs. high |  | 6.2 | **< 0.001 ***** |
|  | Female | Low vs. medium |  | 1.6 | 0.254 |
|  |  | Medium vs. high |  | 0.3 | 0.965 |
|  |  | Low vs. high |  | 1.3 | 0.381 |
|  | Low | Male vs. female |  | 0.0 | 0.975 |
|  | Med | Male vs. female |  | 1.8 | 0.076 |
|  | High | Male vs. female |  | 3.3 | **0.001 **** |

**S5 Table continued.** Social Effort Task Statistics with social anxiety: pairwise comparisons of significant main and interaction effects

|  |  |  | df | *t* | p |
| --- | --- | --- | --- | --- | --- |
| **Social anxiety * Social status** | Non-elevated | Low vs. medium |  | 6.3 | **< 0.001 ***** |
|  |  | Med vs. high |  | 4.0 | **< 0.001 ***** |
|  |  | Low vs. high |  | 10.3 | **< 0.001 ***** |
|  | Elevated | Low vs. medium |  | 1.2 | 0.442 |
|  |  | Med vs. high |  | 0.4 | 0.895 |
|  |  | Low vs. high |  | 0.8 | 0.721 |
|  | Low | Non-elevated vs elevated |  | 1.3 | 0.210 |
|  | Med | Non-elevated vs elevated |  | 0.0 | 0.985 |
|  | High | Non-elevated vs elevated |  | 1.8 | 0.068 |
