# Supplementary material for: An effort-based social feedback paradigm reveals aversion to popularity in socially anxious participants and increased motivation in adolescents
Source: PLoS One. 2021 Apr 27;16(4):e0249326. doi: 10.1371/journal.pone.0249326 (PMC8078767; doi:10.1371/journal.pone.0249326)
Supplement: S6 Table — (DOCX) [file pone.0249326.s008.docx]

**S6 Table.** Social Effort Task Statistics with social anxiety at LSAS cut-off of 47

|  |  | Error df, df | F | p |
| --- | --- | --- | --- | --- |
| **Main Effects** | **Social status** (low/medium/high) | 2, 684 | 33.9 | **< 0.001 ***** |
|  | **Probability** (12%/50%/88%) | 2, 684 | 19.9 | **< 0.001 ***** |
|  | **Social anxiety** (non-elevated/elevated) | 1, 684 | 1.1 | 0.303 |
|  | **Age** (adolescents/young adults) | 1, 81 | 3.0 | 0.087 |
|  | **Sex** (male/female) | 1, 81 | 8.3 | **0.005 **** |
| **Two-Way Interactions** | **Social status x probability** | 4, 684 | 2.7 | **0.030 *** |
|  | **Social status x social anxiety** | 2, 684 | 6.1 | **0.002 **** |
|  | **Social status x age** | 2, 684 | 1.8 | 0.163 |
|  | **Social status x sex** | 2, 684 | 10.5 | **< 0.001 ***** |
|  | **Probability x social anxiety** | 2, 684 | 0.4 | 0.691 |
|  | **Probability x age** | 2, 684 | 2.4 | 0.094 |
|  | **Probability x sex** | 2, 684 | 2.1 | 0.129 |
|  | **Social anxiety x age** | 1, 81 | 0.0 | 0.915 |
|  | **Social anxiety x sex** | 1, 81 | 0.1 | 0.716 |
|  | **Age x sex** | 1, 81 | 0.0 | 0.837 |

Three- and four-way interactions were not significant and dropped from the design.
