# Supplementary material for: An effort-based social feedback paradigm reveals aversion to popularity in socially anxious participants and increased motivation in adolescents
Source: PLoS One. 2021 Apr 27;16(4):e0249326. doi: 10.1371/journal.pone.0249326 (PMC8078767; doi:10.1371/journal.pone.0249326)
Supplement: S7 Table — (DOCX) [file pone.0249326.s009.docx]

**S7 Table.** Social Effort Task Statistics with continuous LSAS and age scores

|  |  | Error df, df | F | p |
| --- | --- | --- | --- | --- |
| **Main Effects** | **Social status** (low/medium/high) | 2, 684 | 7.0 | **< 0.001 ***** |
|  | **Probability** (12%/50%/88%) | 2, 684 | 1.0 | 0.359 |
|  | **Social anxiety** | 1, 684 | 0.0 | 0.872 |
|  | **Age** | 1, 81 | 0.9 | 0.335 |
|  | **Sex** (male/female) | 1, 81 | 0.0 | 0.916 |
| **Two-Way Interactions** | **Social status x probability** | 4, 684 | 2.7 | **0.032 *** |
|  | **Social status x social anxiety** | 2, 684 | 3.3 | **0.035 *** |
|  | **Social status x age** | 2, 684 | 1.2 | 0.302 |
|  | **Social status x sex** | 2, 684 | 10.3 | **< 0.001 ***** |
|  | **Probability x social anxiety** | 2, 684 | 0.3 | 0.722 |
|  | **Probability x age** | 2, 684 | 1.1 | 0.340 |
|  | **Probability x sex** | 2, 684 | 1.9 | 0.148 |
|  | **Social anxiety x age** | 1, 81 | 0.0 | 0.997 |
|  | **Social anxiety x sex** | 1, 81 | 0.1 | 0.750 |
|  | **Age x sex** | 1, 81 | 0.3 | 0.566 |

Three- and four-way interactions were not significant and dropped from the design.
