# Supplementary material for: An effort-based social feedback paradigm reveals aversion to popularity in socially anxious participants and increased motivation in adolescents
Source: PLoS One. 2021 Apr 27;16(4):e0249326. doi: 10.1371/journal.pone.0249326 (PMC8078767; doi:10.1371/journal.pone.0249326)
Supplement: S8 Table — (DOCX) [file pone.0249326.s010.docx]

**S8 Table.** Effects of fatigue

|  |  | Error df, df | F | p |
| --- | --- | --- | --- | --- |
| **Main Effects** | **Fatigue** (begin/mid/end task) | 2, 174 | 21.8 | **< 0.001 ***** |
|  |  | df | *t* | p |
| **Fatigue** | **Begin vs. mid task** |  | 2.2 | 0.072 |
|  | **Begin vs. end task** |  | 6.5 | **< 0.001 ***** |
|  | **Mid vs. end task** |  | 4.3 | **< 0.001 ***** |
